# Role of Basophils in a Broad Spectrum of Disorders

**DOI:** 10.3389/fimmu.2022.902494

**Published:** 2022-05-27

**Authors:** Kensuke Miyake, Junya Ito, Hajime Karasuyama

**Affiliations:** Inflammation, Infection and Immunity Laboratory, Advanced Research Institute, Tokyo Medical and Dental University (TMDU), Tokyo, Japan

**Keywords:** basophils, allergy, IL-4, tissue repair, fibrosis, autoimmune diseases, tumor, COVID-19

## Abstract

Basophils are the rarest granulocytes and have long been overlooked in immunological research due to their rarity and similarities with tissue-resident mast cells. In the last two decades, non-redundant functions of basophils have been clarified or implicated in a broad spectrum of immune responses, particularly by virtue of the development of novel analytical tools for basophils. Basophils infiltrate inflamed tissues of patients with various disorders, even though they circulate in the bloodstream under homeostatic conditions. Depletion of basophils results in the amelioration or exaggeration of inflammation, depending on models of disease, indicating basophils can play either beneficial or deleterious roles in a context-dependent manner. In this review, we summarize the recent findings of basophil pathophysiology under various conditions in mice and humans, including allergy, autoimmunity, tumors, tissue repair, fibrosis, and COVID-19. Further mechanistic studies on basophil biology could lead to the identification of novel biomarkers or therapeutic targets in a broad range of diseases.

## 1 Introduction

Basophils are the least common granulocytes, representing ~0.5% of peripheral blood leukocytes in both mice and humans. Basophils have long been regarded erroneously as the blood-circulating mast cells, due to their phenotypic similarities with tissue-resident mast cells, including the surface expression of the high-affinity IgE receptor (FcεRI), and the release of histamine in response to various stimuli. Actually, in clinical settings, basophils are frequently used as a surrogate for tissue-resident mast cells for allergy diagnosis. Nevertheless, basophils and mast cells differ from each other in several aspects. Basophils usually circulate in the blood, while mast cells reside in peripheral tissues. Basophils have much shorter lifespan than mast cells. Moreover, the gene expression profile is quite distinct between basophils and mast cells in both mice and humans (1, 2), implying that basophils have unique roles distinct from those played by mast cells.

In the last two decades, a series of analytical tools for basophils have been developed, including basophil-depleting antibodies (3, 4), genetically engineered mice which specifically lack basophils (5–11), basophil-reporter mice (7, 11, 12), and basophil-specific Cre-expressing mice (7, 13, 14). Studies using these powerful tools have identified non-redundant roles of basophils in Th2-type immune responses, including the allergic inflammation (15–17) and protective immunity against parasitic infections (18–20). Basophils are also shown to play important roles in other types of responses, such as autoimmunity (21), tissue repair (22), fibrosis (23), cancer (24–26), and possibly COVID-19 pathogenesis (27). In this review, we summarize the recent developments on the contribution of basophils to the pathogenesis of a variety of inflammatory disorders, based on research findings published mainly during the past 5 years. Regarding inflammatory responses associated with parasitic infections, we highly recommend readers to refer recent review articles (18–20).

## 2 Role of Basophils in Allergic Inflammation

### 2.1 Basophils as a Tool for Allergy Diagnosis

Basophils isolated from patients’ blood are often used for allergy testing in clinical settings. The basophil activation test (BAT) is a representative assay, in which patient’s blood is incubated with suspected allergens, and the activation of basophils in the blood is assessed by the upregulation of CD63 and/or CD203c on their cell surface (28, 29). BAT is useful for the diagnosis of a wide variety of allergic disorders, including allergy to food, drug, or venom as well as allergic rhinitis and asthma. BAT is also utilized to monitor allergy therapeutics, such as allergen immunotherapy and anti-IgE therapy (30–32).

Approximately 10-15% of individuals have basophils that are non-responsive to anti-IgE antibody or allergen stimulation, known as non-responder or non-releaser basophils (33). The non-responder basophil phenotype is associated with downregulation of spleen tyrosine kinase (Syk), even though the functional significance of non-responder basophils remains elusive. The presence of non-responder basophils is a challenge when using BAT for allergy diagnosis.

BAT is a potential diagnostic tool for hypersensitivity reactions against COVID-19 mRNA vaccines, but its usefulness for predicting allergic reactions to the mRNA vaccines remains controversial. Troelnikov et al. recruited three patients with a history of polyethylene glycol (PEG) allergy and found that all three patients displayed a positive skin intradermal test and BAT for a PEG-containing mRNA vaccine, while all of them displayed negative BAT for PEG itself (34). Basophils from these patients were also activated by PEGylated liposomal doxorubicin, suggesting that PEGylated lipid nanoparticles, but not PEG itself, are the cause of their hypersensitivity reactions. Warren et al. recruited patients with a previous history of allergic reactions against mRNA vaccines (35). Only 1 of 11 patients displayed a positive skin prick test for the mRNA vaccines, even though all patients clinically underwent allergic reactions to the mRNA vaccines. In contrast, 10 of 11 patients displayed a positive BAT against PEG alone while all 11 patients had a positive BAT against the mRNA vaccines, even though PEG-specific IgE could not be detected in these patients. This suggests a role for non-IgE-mediated allergic reactions against the COVID-19 mRNA vaccines. Interestingly, Labella et al. reported that 50% of persons with a history of SARS-CoV-2 infection displayed positive BAT against the mRNA vaccine, irrespective of their vaccination status (36). Therefore, a positive BAT result against the mRNA vaccines may be attributed to either a PEG allergy or a previous SARS-CoV2 infection.

Additional allergy tests using basophils have been proposed. McKenzie et al. established a method for detecting allergen specific IgE on basophils, designated as CytoBas (37). Qi et al. reported that upon activation, basophils and mast cells release CD203c^+^ extracellular vesicles, and the presence of such vesicles has strong diagnostic value in patients with drug allergies (38).

### 2.2 Skin Allergy

#### 2.2.1 Cutaneous Basophils Hypersensitivity

In the 1970s, basophils attracted attention since massive infiltration of basophils into the skin lesion was observed in certain forms of delayed-type hypersensitivity reactions triggered by the injection of foreign antigens (39, 40). It is called cutaneous basophil hypersensitivity (CBH) and mainly studied in guinea pigs. This reaction clinically and histologically resembles Jones-Mote responses to rabbit serum proteins in humans. CBH is hardly elicited in mice, and the functional role of basophils in CBH remains to be clarified. Skin allergic reactions against COVID-19 mRNA vaccination clinically resemble Jones-Mote reactions (41) characterized by erythematous and indurated skin reactions. Although it remains unclear whether basophils are indeed recruited to the site of the mRNA vaccination, it would be possible that basophils are potentially involved in the hypersensitivity reactions against COVID-19 mRNA vaccines since some reports indicate the IgE-independent basophil activation by mRNA vaccines (34, 35).

#### 2.2.2 Atopic Dermatitis

**Figure 1:**
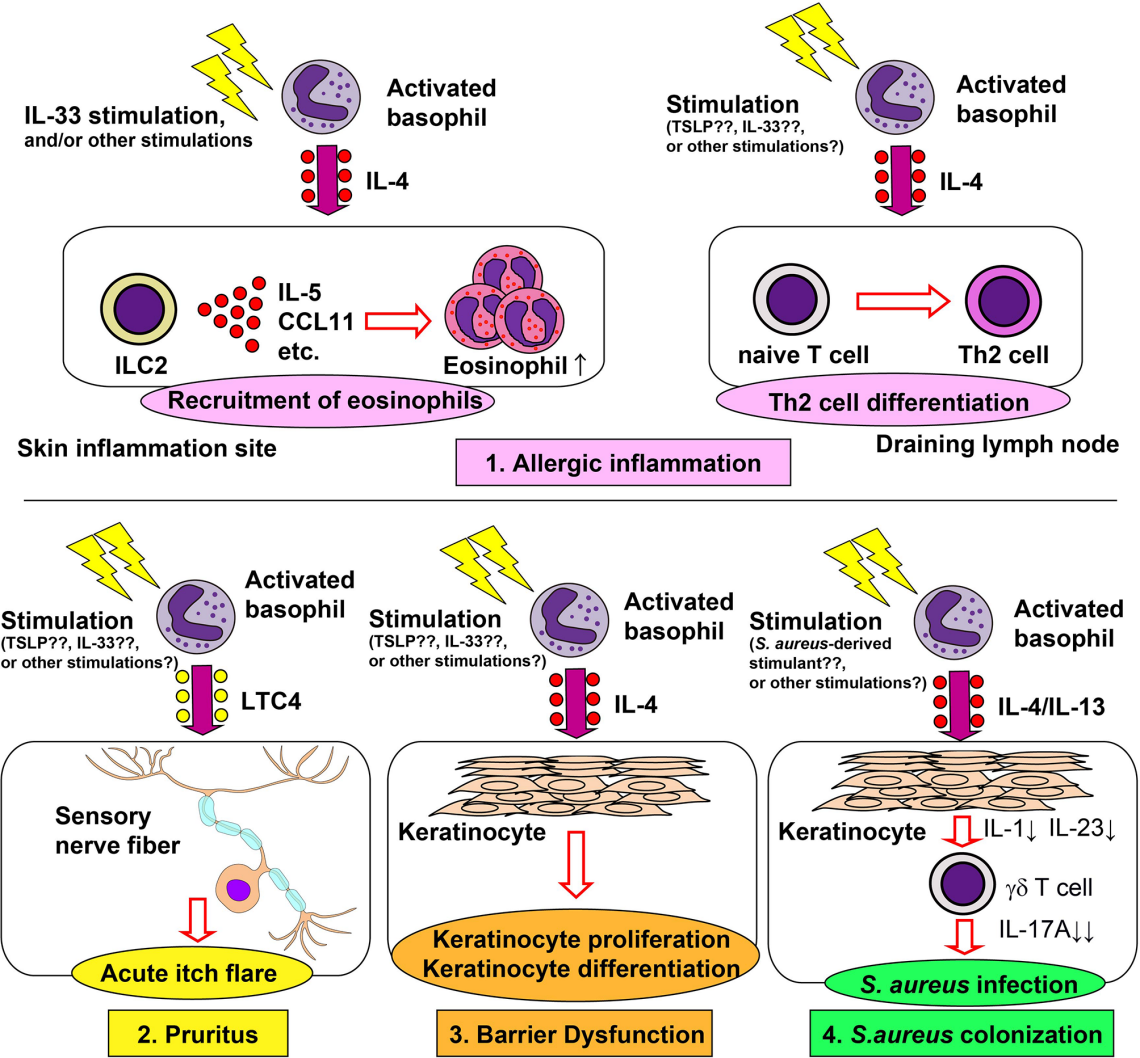
Role of basophils in the pathogenesis of atopic dermatitis. Basophils contribute to allergic inflammation, pruritus, barrier dysfunction and *Staphylococcus aureus* colonization in atopic dermatitis (AD). 1) Basophil-derived interleukin (IL)-4 stimulates group 2 innate lymphoid cells (ILC2s) to enhance the production of IL-5 and CCL11, leading to enhanced recruitment of eosinophils to the skin lesion (upper left panel). Basophil-derived IL-4 also augments the differentiation of naïve T cells into Th2 cells in draining lymph nodes (upper right panel). 2) Basophil-derived leukotriene C4 (LTC4) acts on CysLTR2^+^ sensory neurons, inducing acute itch flares in AD (lower left panel). 3) Basophil-derived IL-4 promotes proliferation and differentiation of keratinocyte (lower middle panel). 4) Basophil-derived IL-4 suppresses the IL-1 and IL-23 production by keratinocytes, leading to reduced production of IL-17A by γδ T cells and, therefore, increased susceptibility to *S. aureus* infection (lower right panel).

The infiltration of basophils in the skin has been described in several inflammatory skin disorders, including atopic dermatitis (AD), bullous pemphigoid, prurigo, Henoch-Shönlein purpura, eosinophilic pustular folliculitis (Ofuji’s disease), and urticaria (42, 43). Of note, peripheral blood basophils from patients with AD display upregulated expression of activation markers such as CD63 and CD203c on the cell surface, compared with those from healthy controls (44, 45). This suggests a possible role for basophils in the pathogenesis of AD. As discussed below, studies from mouse AD models demonstrated that basophils contribute to the allergic inflammation, pruritus, and barrier dysfunction in AD, three key features that contribute to the pathogenesis of AD (46) (**Figure 1**).

##### 2.2.2.1 Allergic Inflammation in AD

Basophils play important roles in the induction of skin allergic inflammation in multiple models of AD, including IgE-dependent chronic allergic inflammation (IgE-CAI) (47, 48), the MC903-induced model (42), the oxazolone-induced model (49, 50), and the IL-33-transgenic mouse model (51). In IgE-CAI, mice are first sensitized with hapten trinitrophenol-specific IgE, followed by an intradermal challenge of corresponding allergens in ear skin, which results in severe ear swelling and infiltration of inflammatory cells, including eosinophils, macrophages, neutrophils, and basophils. IgE-CAI can be elicited even in the absence of mast cells. On the other hand, depletion of basophils almost completely abolishes ear swelling and cellular infiltration (3, 6, 48), illustrating an essential role for basophils in the induction of IgE-CAI. Serine proteases released by basophils play critical roles in the development of IgE-CAI (52). Moreover, basophil-derived interleukin (IL)-4 plays a key role in the recruitment of eosinophils to the skin lesion, and thus promotes cutaneous inflammation (53). In an IgE-dependent skin allergic inflammation model similar to IgE-CAI, basophil-derived IL-4 promotes the expression of adhesion molecules, resulting in the enhanced recruitment of eosinophils (54). Basophil-derived IL-4 also plays an important role in the development of AD induced by repetitive topical application of hapten oxazolone (50). In this model, basophils are the major source of IL-4 in the skin lesion, consistent with recent single-cell RNA-seq data (55). Depletion of basophils ameliorates eczematous skin inflammation with crusts and scales, suggesting the role of basophil-derived IL-4 in the formation of lichenized skin lesions. Basophil-derived IL-4 also plays key roles in other AD models. In a topical MC903 application model, basophil-derived IL-4 acts on skin-resident group 2 innate lymphoid cells (ILC2s), leading to enhanced proliferation of ILC2s and AD-like skin inflammation (42). Similarly, in the IL-33-transgenic mouse model, basophils promote the proliferation of ILC2s, possibly through the production of IL-4 (51). Thus, basophil-derived IL-4 appears to contribute to AD pathogenesis in the mouse models. In moderate-to-severe AD patients, the treatment with dupilumab, a human monoclonal antibody against IL-4Rα, rapidly improves the disease (56), suggesting the involvement of IL-4 and IL-13 in the pathogenesis of human AD. Given that human basophils can produce a large amount of IL-4 in response to various stimuli (57), it is likely that basophils contribute to the pathogenesis of certain types of AD in humans.

Several reports indicate that basophils contribute to Th2 cell differentiation in mouse AD models (12, 49, 58). Infiltration of basophils into skin draining lymph nodes is observed in some AD models, including the MC903-induced and oxazolone-induced models (12, 49), suggesting that basophils can provide IL-4 required for the differentiation of naive CD4^+^ T cells into Th2 cells. Moreover, basophils are capable of presenting antigens to naïve T cells, leading to the induction of Th2 cell differentiation (59–61). However, this role of basophils remains controversial, since some reports argued that basophils are incapable of processing and presenting antigens to naïve T cells (62, 63). Miyake et al. revisited this issue and found that basophils acquire peptide-MHC-II complexes from dendritic cells (DCs) through trogocytosis and can present antigens to naïve T cells, promoting their differentiation into Th2 cells (35). Therefore, the functional significance of basophils in Th2 cell differentiation may differ, depending on experimental settings, determined in part by the extent of the basophil-DC interaction in draining lymph nodes.

##### 2.2.2.2 Pruritus in AD

AD is characterized by chronic and intense itch, which can be mediated by both histaminergic and non-histaminergic pathways (64). Th2 cytokines including IL-4, IL-13, and IL-31 interact with sensory neurons to provoke chronic itch in the context of AD (65, 66). Basophils can produce a large amount of IL-4 in response to various stimuli (57), and are the major source of IL-4 in the skin lesions of multiple AD models (48, 50, 67). Moreover, basophils can produce IL-31 in response to anti-IgE and N-formyl-methionyl-leucyl-phenylalanine stimulation (68). Therefore, it is probable that basophils play a role in pruritus in AD.

Approximately 50% of patients with AD experience acute itch flares, which is the exacerbation of intense itch, within 2 months. Wang et al. showed that basophils contribute to acute itch flares by interacting with sensory neurons *via* leukotriene C4 (LTC4) in a mouse model. Mice were first topically sensitized with MC903 and allergens (ovalbumin; OVA) on ear skin for 10 days, followed by an intradermal OVA challenge at a separate skin site, leading to acute itch flares at the challenge site, and chronic itch at the sensitization site (44). Basophil depletion significantly reduced the occurrence of acute itch flares, whereas mast cell-deficiency had no effect. Moreover, chemogenic activation of basophils induced acute itch flares, suggesting the critical roles of basophils in acute itch flares. Importantly, pharmacological inhibition of LTC4 significantly reduced the occurrence of acute itch flares, and LTC4 levels in the skin lesion were partly dependent on basophils. These results indicate the possible involvement of the basophil-LTC4 axis in acute itch flares.

##### 2.2.2.3 Skin Barrier Dysfunction in AD

Skin barrier dysfunction is considered critical for the pathogenesis of AD. Damaged epithelial cells produce cytokines such as thymic stromal lymphopoietin (TSLP) and IL-33, leading to the promotion of Th2 immunity (69). Th2 immunity in turn promotes further disruption of the skin barrier (70). Consistent with this, the *in vitro* treatment of keratinocytes with either IL-4 or IL-13 induced the reduction of barrier-related genes (71, 72). Of note, the treatment of AD patients with dupilumab significantly increased epidermal barrier-related genes (73), suggesting that IL-4 and/or IL-13 promote skin barrier dysfunction in AD patients. In accordance with this, in a MC903-induced AD model, basophil depletion decreased the expression of IL-4 and IL-13 in skin lesion, reduced epidermal hyperplasia and keratinocyte proliferation and significantly reduced trans-epidermal water loss (67), indicating the involvement of basophils in skin barrier dysfunction through the IL-4 and IL-13 production.

##### 2.2.2.4 Staphylococcus aureus Infection in AD

Skin colonization of *S. aureus* is frequently observed in patients with AD and is involved in the pathogenesis of AD (74, 75). In mice, the intradermal injection of lipoteichoic acid, a principal cell wall component of *S. aureus*, enhances skin recruitment of basophils in a TSLP-dependent manner (76). Moreover, basophil-derived IL-4 promoted cutaneous *S. aureus* infection in a mouse model of *S. aureus* infection. The tape stripping-induced skin barrier disruption triggered the recruitment of basophils to the skin and facilitated cutaneous *S. aureus* colonization (77). Either basophil depletion or basophil-specific IL-4/IL-13 deficiency protected mice from enhanced *S. aureus* infection. Basophil-derived IL-4/IL-13 suppresses IL-1 and IL-23 production by keratinocytes. This leads to reduced IL-17A expression by γδ T cells and impaired production of neutrophil-attracting chemokines in the skin, resulting in enhanced *S. aureus* infection. Callewaert et al. reported that the dupilumab treatment reduces *S. aureus* colonization in patients with AD (78), suggesting that basophil-derived IL-4/IL-13 may also promote cutaneous *S. aureus* infections in AD patients.

##### 2.2.2.5 Resolution of AD

Basophils reportedly contribute to the resolution of allergic inflammation through the generation of M2 macrophages. In the IgE-CAI model, basophil-derived IL-4 promotes the differentiation of inflammatory monocytes into anti-inflammatory M2 macrophages, leading to the resolution of skin allergic inflammation (48). Pellefigues et al. identified two distinct roles for basophils during an allergic inflammation in another AD model, in which mice were topically applied with MC903 for 4 consecutive days only. Mice developed continual aggravation of ear swelling and skin barrier dysfunction for approximately 9-10 days (inflammation phase), followed by the resolution of skin inflammation after 10 days (resolution phase). Depletion of basophils in the inflammation phase resulted in the improvement of barrier dysfunction, indicating the proinflammatory role of basophils. By contrast, either basophil-specific macrophage colony-stimulating factor (M-CSF)-deficiency or IL-4 neutralization resulted in aggravated ear swelling and skin barrier dysfunction in the resolution phase. Considering that IL-4 and M-CSF are important for the generation of anti-inflammatory M2 macrophages, basophil-derived IL-4 and M-CSF likely cooperate together to promote M2 macrophage differentiation, leading to the resolution of AD. In line with this, basophil-depletion at the resolution phase resulted in the impaired generation of CD206^hi^ macrophages and reduced efferocytosis (phagocytosis of apoptotic cells) capacity in macrophages.

#### 2.2.3 Chronic Spontaneous Urticaria

Chronic spontaneous urticaria (CSU) is characterized by itchy hives or angioedema which lasts for at least 6 weeks. Several lines of evidence have suggested the role of basophils in the pathogenesis of CSU (79–81). Ito et al. demonstrated the recruitment of basophils to skin lesions in patients with CSU (43). Extreme basopenia in the blood is commonly observed in patients with CSU (82, 83), and blood basopenia is associated with disease severity (84, 85). Omalizumab, a humanized monoclonal anti-IgE antibody, has been approved for the treatment of CSU (86, 87). Notably, the omalizumab therapy rapidly increases the blood basophil number (88–90), whereas the number of FcεRI^+^ cells in skin lesions is decreased by omalizumab treatment (88). These results indicate the possibility that blood basopenia mirrors the recruitment of basophils to the skin lesion.

In some patients with CSU, IgG autoantibodies against IgE and FcεRI are detected (91–93), which is considered to be a cause of CSU (94). A recent study revealed that blood basopenia is strongly associated with the presence of autoantibodies against IgE or FcεRI (95). Furthermore, blood basopenia combined with autoantibodies is a predictor for slower response to omalizumab therapy (96). Nonetheless, how basopenia is associated with autoantibody and poor therapeutic responses against omalizumab remains unclear. Autoantibody-mediated activation of basophils may promote basophil recruitment to the skin lesion resulting in basopenia in the blood. It is also postulated that patients bearing autoantibodies display resistance to omalizumab therapy, possibly due to the inability of anti-IgE antibody to interfere with IgG autoantibody-mediated basophil activation.

### 2.3 Respiratory Allergy

In patients with moderate-to-severe asthma, the treatment with dupilumab, an anti-IL-4Rα antibody, efficiently reduces severe exacerbation and improves lung functions (97), suggesting the involvement of IL-4 and/or IL-13 in the pathogenesis of asthma. In line with this, several animal studies demonstrated the therapeutic effect of IL-4 and IL-13 inhibition on type 2 lung inflammation (98–100). Basophils are a potent source of IL-4 and IL-13 (50) and therefore may contribute to the development of lung inflammation in patients with asthma. Indeed, histopathological analysis revealed basophil infiltration in the lungs of asthmatic patients, especially in fatal cases (101, 102). Sputum basophils from asthmatic patients display increased expression of activation markers such as CD63 and CD203c, compared with blood basophils (103). Notably, the treatment of asthma patients with benralizumab, a human monoclonal antibody against IL-5Rα, significantly reduces the count of not only eosinophils but also basophils in the blood (104–106). This suggests that the therapeutic effect of benralizumab can be attributed in part to the basophil depletion.

The frequency of basophils in the sputum of patients is higher in eosinophilic asthma than in non-eosinophilic asthma and healthy controls (103, 107) and positively correlated with that of sputum eosinophils, implying a possible role of basophils in eosinophil recruitment to inflamed lungs. In accordance with this, the expression of basophil/mast cell-related genes in sputum is associated with lung eosinophilic inflammation (108). In a papain-induced asthma model, activated basophils produce IL-4 which promotes the proliferation of ILC2, enhances the production of IL-5 and CCL11 from TSLP-activated ILC2s, and facilitates recruitment of eosinophils into the lungs (109, 110). Matsuyama et al. reported that the long-acting muscarinic antagonist acts on muscarinic M3 receptor expressed by basophils to suppress IL-4 production. Therefore, it inhibits activation and proliferation of ILC2s, leading to the reduced eosinophil infiltration and airway inflammation. Unlike basophils, mast cells rather suppress papain-induced lung allergic inflammation by promoting regulatory T cell expansion (111).

Infiltration of basophils is observed in nasal polyps in patients with chronic rhinosinusitis (112–114). Recent study suggested the role of basophils in aspirin-exacerbated respiratory disease (AERD) (114), which is characterized by the triad of chronic rhinosinusitis, namely nasal polyps (CRSwNP), asthma and intolerance to cyclooxygenase-1 inhibitors. Patients with AERD displayed increased basophil numbers in nasal polyps and peripheral blood, compared to patients with CRSwNP alone. Basophils from nasal polyps of AERD patients displayed an activated phenotype and increased rates of degranulation, as assessed by the loss of staining with 2D7, a basophil granule-specific antibody. Frequency of basophil degranulation was positively correlated with disease severity, suggesting the possible contribution of basophils to the pathogenesis of AERD.

### 2.4 Gastrointestinal Allergy

As mentioned earlier, basophils are extensively utilized for the diagnosis of food allergies. Besides their usefulness in the diagnosis, basophils are implicated in the pathogenesis of peanut allergy in a human study (115). In mouse models of epicutaneous allergen sensitization, basophils promote the production of allergen-specific IgE, leading to the development of food allergies (116–119). Basophils also play a key role in the effector phase of food allergies (120). After repeated intragastric challenge with allergens, basophils infiltrated the jejunum, and the depletion of basophils reduced the incidence of diarrhea, concurrent with the reduction of mMCP-1^+^ mast cells in the jejunum. In this model, IL-4 produced by basophils plays a key role in the food allergy pathogenesis. Given that IL-4Rα on mast cells plays a critical role in this model (121), it can be assumed that basophil-derived IL-4 acts on mast cells to aggravate food allergies.

Recent studies have identified the role of basophils in the pathogenesis of eosinophilic esophagitis (EoE), a food allergy-related chronic and inflammatory esophageal disorder (122, 123). Infiltration of basophils into the esophagus is observed in patients with active EoE, and the frequency of basophils in the esophagus is positively correlated with eosinophil infiltration in the esophagus (122). In mice, EoE-like esophageal inflammation can be triggered by epicutaneous sensitization and subsequent challenge with food allergens (122, 123). Basophil depletion or TSLP neutralization significantly reduces the esophageal infiltration of eosinophils, highlighting the roles of TSLP and basophils in the pathogenesis of the mouse EoE model. In another mouse model of EoE, the IL-33 receptor on basophils plays a key role in the recruitment of basophils to the esophagus and esophageal eosinophilic inflammation (123).

### 2.5 Systemic Anaphylaxis

Basophils reportedly contribute to IgG1-mediated anaphylaxis through the release of the platelet-activating factor (PAF) in a mouse model (124). In a food allergy model, mice deficient for the inhibitory receptor Allergin-1 showed systemic anaphylaxis in a basophil-dependent manner (125). Human studies also indicated the possible involvement of basophils in systemic anaphylaxis. In patients with acute anaphylaxis, blood basophil numbers decreased in the acute phase and recovered in the convalescent phase (126–128). Concurrently, the intracellular histamine content of basophils significantly decreased in patients with anaphylaxis (128). Serum levels of CCL2 significantly increased in the acute phase, as compared with healthy controls or with the convalescent phase (126, 127). Further *in vitro* experiments revealed that the serum from patients with acute phase anaphylaxis promoted basophil chemotaxis in a CCL2-depedent manner, suggesting the role of CCL2-mediated basophil chemotaxis during systemic anaphylaxis (127).

### 2.6 Functional Significance of CD15s Expressed on Human Basophils in Allergic Inflammation

Puan et al. reported that single nucleotide polymorphisms (SNPs) in the fucosyltransferase 6 (*FUT6*) gene locus are associated with the surface expression of CD15s (sialyl Lewis x) in human basophils (129). *In vitro* experiments showed that CD15s on basophils is functionally important for the rolling on E-selectin-coated surfaces. Indeed, patients with *FUT6* null mutations had higher basophil numbers in the blood and lower itch sensitivity against mosquito bites. Furthermore, *FUT6*-deficiency significantly reduced serum concentration of total IgE and house dust mite (HDM)-specific IgE and decreased skin prick responses against HDM antigens. These results highlight the role of basophils in HDM-induced allergy and itch sensitivity against insect bites.

## 3 Role of Basophils in Tissue Repair and Fibrosis

### 3.1 Resolution of Inflammation and Tissue Repair

**Figure 2:**
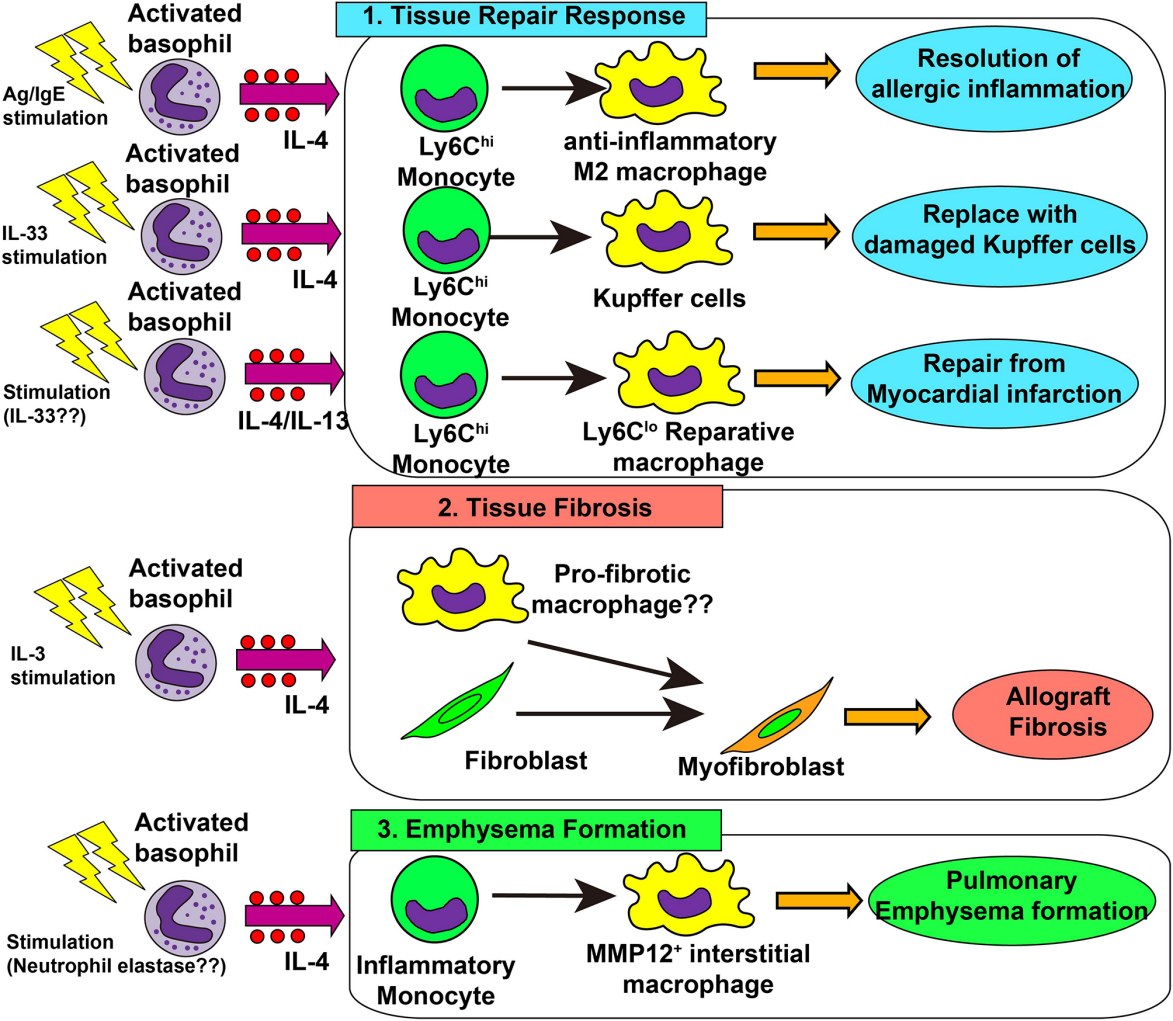
Role of basophils in tissue repair responses, tissue fibrosis and emphysema formation. 1) In skin allergy, basophil-derived interleukin (IL)-4 promotes the differentiation of inflammatory monocytes into anti-inflammatory M2 macrophages, leading to the resolution of allergic inflammation. In the liver infected with *Listeria monocytogenes*, basophil-derived IL-4 promotes monocyte differentiation into M2 macrophages which replace damaged Kupffer cells, promoting healing of liver damage. In myocardial infarction, basophil-derived IL-4 and/or IL-13 promotes the generation of reparative Ly6C^lo^ macrophages which enhances post-infarction tissue repair. 2) In allograft heart transplantation, basophil-derived IL-4 promotes tissue fibrosis, possibly by acting on tissue-resident macrophages or fibroblasts, leading to the generation of myofibroblasts and deposition of collagen fibers. 3) In a chronic obstructive lung disease (COPD) model, basophil-derived IL-4 promotes the generation of MMP-12^+^ interstitial macrophages which promote the destruction of alveolar walls and emphysema formation.

Following acute and chronic inflammation, an integrated resolution process takes place, which results in the reduction of cellular infiltration and tissue damage repair (130). As mentioned earlier, basophils promote the generation of anti-inflammatory M2 macrophages, leading to the resolution of allergic inflammation (48, 67). This beneficial role of basophils was also demonstrated in liver tissue repair after *Listeria monocytogenes* (Lm) infection (131). Lm infection induced necroptosis of liver-resident Kupffer cells, which triggered IL-33 production from hepatocytes and IL-4 production from recruited basophils. Recruited monocytes were alternatively activated by basophil-derived IL-4, leading to the replacement of ablated Kupffer cells with monocyte-derived macrophages. This was also the case when healing after myocardial infarction (MI) (22). In an animal model of MI, basophils infiltrated heart tissue, and basophil depletion or basophil-specific IL-4/IL-13-deficiency resulted in the deterioration of cardiac functions and increased the infarct region. Moreover, basophil-specific IL-4/IL-13-deficiency increased the infiltration of inflammatory Ly6C^hi^ monocytes and reduced Ly6C^lo^ reparative macrophage numbers, suggesting the involvement of basophil-derived IL-4/IL-13 in the phenotypic transition from inflammatory monocytes to reparative macrophages (22, 132). Stimulation of basophils by IPSE/α-1 significantly enhanced cardiac functions, demonstrating the important role of activated basophils in post-MI tissue repair. In patients with acute MI, low blood basophil counts were associated with increased scar size and poor outcomes, which raises the possibility that basophils contribute to the tissue repair process. Thus, basophil-derived IL-4 and IL-13 contributes to a variety of tissue repair responses (**Figure 2** upper panel).

Basophil-derived amphiregulin is also involved in UVB-induced suppression of cutaneous inflammation (133). Inclan-Rico et al. revealed that basophils contributed to the resolution of lung inflammation by inhibiting ILC2 activation in a helminth infection model (134). Lung-infiltrated basophils stimulate ILC2s to enhance the expression of the receptor for the neuropeptide neuromedin B, and neuromedin B-mediated signals inhibit exaggerated activation of ILC2s and prevent excess lung inflammation.

### 3.2 Tissue Fibrosis and Emphysema

Type 2 immunity promotes tissue repair whereas the dysregulated or chronic tissue repair program leads to tissue fibrosis (135). In an allograft heart transplantation model, basophil-derived IL-4 was involved in tissue fibrosis (23). Donor-derived basophils infiltrated allograft heart and produced IL-4, while basophil depletion reduced the number of α-SMA^+^ myofibroblasts and hence inhibits allograft fibrosis. In accordance with this, an IL-4 receptor-deficient graft heart was resistant to tissue fibrosis. In this model, IL-3 played a role in the recruitment and activation of basophils (136). Therefore, it can be assumed that IL-3-activated basophils produce IL-4 which in turn acts on heart-resident macrophages and fibroblasts to promote the generation of myofibroblasts and deposition of collagens (**Figure 2** middle panel).

A recent report implicated basophils in IgG4-related diseases (IgG4-RD) (137). IgG4-RD is characterized by elevated IgG4 levels in serum, storiform fibrosis, and marked infiltration of IgG4-producing plasma cells in multiple organs, including the pancreas, kidney, and salivary glands. Basophils expressing both TLR2 and TLR4 infiltrated pancreatic tissues in patients with type 1 autoimmune pancreatitis, a pancreatic manifestation of IgG4-RD (138). Furthermore, LPS-stimulated basophils produced IL-13 and B cell-activating factor (BAFF), which induced IgG4 production by B cells (139), suggesting that basophils may contribute to IgG4 production in patients with IgG4-RD.

Dysregulated tissue repair responses can cause pulmonary emphysema (140). Shibata et al. revealed that basophils contribute to the pathogenesis of pulmonary emphysema in an elastase-induced mouse model of chronic obstructive lung disease (COPD) (13). Basophil-derived IL-4 promoted the generation of emphysema-prone MMP12^+^ interstitial macrophages, contributing to lung emphysema formation (**Figure 2** lower panel). On the other hand, genetically engineered mast cell-deficient mice (*Cpa3* ^Cre/+^ mice) developed pulmonary emphysema as much as observed in mast cell-sufficient mice, even though these mice showed partial reduction of basophils (141). A recent report showed basophil infiltration in the inflamed lung tissue of patients with COPD, especially in severe cases (142), suggesting the possible contribution of basophils for COPD development in humans.

## 4 Role of Basophils in Autoimmune Diseases

### 4.1 Systemic Lupus Erythematosus

**Figure 3:**
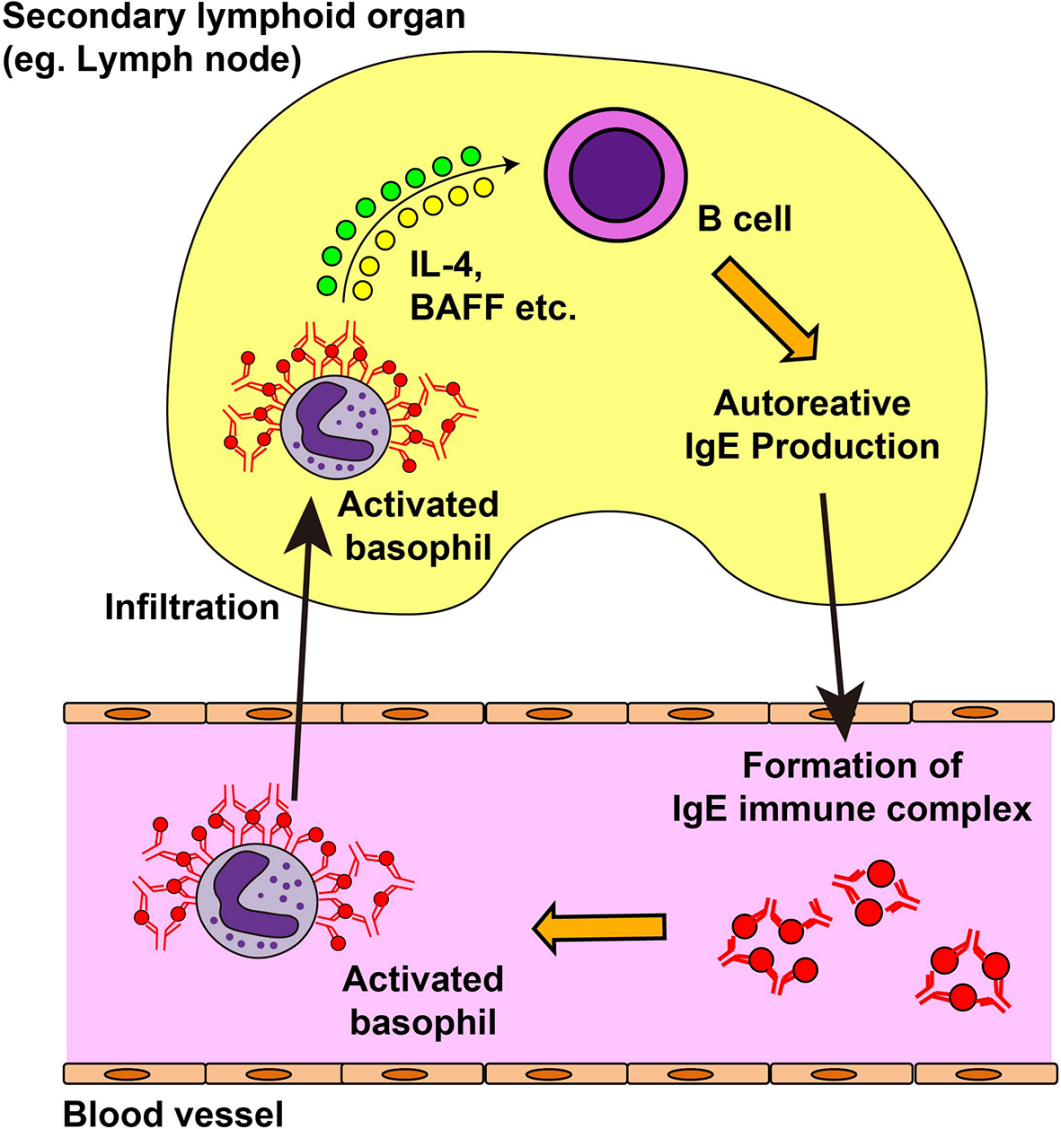
Feedback loop for the exacerbation of systemic lupus erythematosus. In the mouse model of systemic lupus erythematosus (SLE) and in patients with SLE, autoreactive IgE antibodies can be detected in the blood. Autoreactive IgE antibodies form immune complexes with corresponding autoantigens and activate blood-circulating basophils. Activated basophils upregulate the surface expression of CD62L and CXCR4 and infiltrate into lymph nodes. They produce interleukin (IL)-4, IL-13, and B cell-activating factor (BAFF) which in turn induces autoantibody production from B cells, resulting in the deposit of immune complexes in the kidney and in disease progression.

Autoreactive IgE antibodies are frequently detected in patients with systemic lupus erythematosus (SLE), and the serum level of autoreactive IgE is associated with disease activity and active nephritis (143–147). In phase Ib clinical trial, omalizumab therapy showed some efficacy on disease activity in SLE patients with elevated level of serum autoreactive IgE antibody (148), which indicates the role of autoreactive IgE in the pathogenesis of SLE. Moreover, patients with SLE displayed blood basopenia, and their basophils had upregulated expression of activation markers such as CD63 and CD203c, compared with basophils from healthy controls (144, 149). Additionally, patient serum induced basophil activation in an IgE-dependent manner (149). These observations suggest the role of autoreactive IgE antibodies and basophils in SLE. In line with this, basophils play critical roles in animal models of lupus nephritis, including *Lyn*-deficient mice, MRL-*lpr/lpr* lupus prone mice, and a pristane-induced SLE model (144, 149, 150). The presence of a feedback-loop is proposed for the pathogenesis of mouse SLE models and patients (149, 151) (**Figure 3**). In SLE models, basophils are recruited to secondary lymphoid organs, display an activated phenotype, and produce IL-4, IL-13, and BAFF to promote autoantibody production. The formation of immune complexes of IgE autoantibodies and autoantigens further activates blood-circulating basophils to enhance the expression of CD62L, which in turn facilitates the homing of basophils into secondary lymphoid organs. Prostaglandin D2 (PGD2) is involved in the recruitment of basophils to secondary lymphoid organs (152). PGD2, possibly produced by activated basophils, enhances surface expression of CXCR4 in basophils, promoting the migration of basophils into secondary lymphoid organs. In accordance with this, basophils from patients with SLE display high CXCR4 expression on their cell surface, which is positively correlated with disease activity.

### 4.2 Other Autoimmune Diseases

Lamri et al. reported that basophils contributed to the pathogenesis of mixed connective tissue disease (MCTD) (153). Patients with MCTD displayed blood basopenia and upregulated expression of CD203c, CD63 and CXCR4 on the surface of basophils. In a mouse MCTD-like lung inflammation model, the infiltration of activated basophils was observed in lung draining lymph nodes. Depletion of basophils or IgE-deficiency prevented the development of lung MCTD-like inflammation. Yuk et al. showed that basophil-derived IL-6 potentiated T cell differentiation into Th17 cells, thus promoting the development of experimental autoimmune encephalomyelitis (154).

## 5 Role of Basophils in Cancer

Several lines of evidence have indicated the possible role of basophils in the development of cancer (24). In pancreatic ductal adenocarcinoma (PDAC), basophils infiltrate tumor draining lymph nodes and express *IL4*. A higher number of infiltrating basophils is associated with reduced PDAC patient survival. In a mouse model of pancreatic cancer, basophils recruited to tumor draining lymph nodes were activated by T cell-derived IL-3 to produce IL-4, promoting Th2 cell and M2 macrophage differentiation which favors pancreatic cancer development. Similarly, basophil infiltration in the tumor microenvironment was a predictor for poor human gastric cancer prognosis (155).

Basophils reportedly contribute to the pathogenesis of chronic myeloid leukemia (CML) (25). Blood basophilia is frequently observed in patients with CML and is associated with poor prognosis of CML. In a mouse model of CML, basophils produced large amount of CCL3 in the bone marrow, which inhibits the functions of normal hematopoietic stem cells and supports the proliferation of leukemia cells (156).

Intriguingly, basophils played a rather protective role in a mouse melanoma model. The depletion of regulatory T cells resulted in substantial infiltration of basophils and CD8^+^ T cells in the tumor microenvironment achieving complete rejection of transplanted melanoma (157). Tumor-infiltrating basophils produce CCL3 and CCL4 to promote the recruitment of CD8^+^ T cells into the tumor microenvironment enhancing tumor rejection. In patients with ovarian cancer, the activated basophil phenotype was predicative of better prognosis (158).

Taken together, these data show that basophils play either deleterious or protective roles in a context-dependent manner. In accordance with this, the opposite roles of basophils were reported in mutagen-induced skin carcinogenesis in mice. On one hand, Crawford et al. demonstrated the protective role of basophils (159). Repetitive application of DNA-damaging xenobiotics DMBA resulted in the production of autoreactive IgE and accumulation of IgE^+^ basophils in the skin. Deficiency of IgE or FcεRIα promotes tumorigenesis and tumor growth, suggesting the contribution of basophils in the inhibition of epithelial carcinogenesis. On the other hand, Hayes et al. showed that basophils and IgE rather enhanced tumor growth in an inflammation-induced skin carcinogenesis model (160) in which mice were first topically treated with DMBA and subsequently with TPA (protein kinase C activator). This led to the production of natural IgE and the accumulation of IgE^+^ basophils in the skin. IgE-deficiency or basophil depletion abolished epithelial hyperplasia. Basophil-derived histamine is thought to promote epithelial hyperplasia, thus promoting TPA-induced tumor growth.

## 6 Possible Roles of Basophils in COVID-19

Emerging evidence suggests a possible role for basophils in COVID-19 (27). Mass-cytometry analysis revealed that the blood basophil number decreases during the acute phase and is restored in the recovery phase (161). In line with this, several studies demonstrated that the blood basophil number is significantly lower in severe COVID-19 patients, compared with less-severe patients or non-COVID-19 patients (162–167). Therefore, lower blood basophil counts would be a risk factor predicting a poor COVID-19 prognosis (162, 168). Notably, the plasma level of anti-SARS-CoV2 IgG correlated positively with the number of blood-circulating basophils (161), suggesting the possible role for basophils in IgG responses against SARS-CoV2.

Surface expression profiles of blood basophils are also altered in COVID-19 patients, especially in severe cases (164, 167). Basophils showed upregulated expression of activation markers such as CD63 and CD11b in severe cases (167). In line with this, *in vitro* co-culture of basophils with SARS-CoV2 led to the production of IL-4 and IL-13 by basophils (169). Further mechanistic studies will be required to identify whether basophils play protective or deleterious roles in COVID-19.

## 7 Conclusion and Perspectives

Basophils have long been neglected from immunological studies, partly due to their similarity to tissue-resident mast cells, even though they have been evolutionally conserved in many animal species. We now appreciate that basophils have non-redundant roles distinct from those played by mast cells in a variety of immune and inflammatory responses, including IgE-CAI, papain-induced asthma, and elastase-induced COPD models. Studies of animal models identified that basophils infiltrate either the sites of inflammation or the draining lymph nodes and regulate immune responses by interacting with various types of cells, including macrophages, T cells, ILCs and sensory neurons. Recent human studies, including the treatments of patients with therapeutic antibodies (e.g. omalizumab, benralizumab and dupilumab), have advanced our understanding of human basophil pathophysiology. Further mechanistic studies would identify novel roles for basophils in an even broader range of disorders and promote the development of novel strategies for the treatment of such diseases by targeting basophils and their products.

## Author Contributions

All authors listed have made a substantial, direct and intellectual contribution to the work and approved it for publication.

## Funding

This work was supported by research grants from the Japanese Ministry of Education, Culture, Sports, Science and Technology [20K16277(KM), 19H01025 (HK), 22K07115 (KM), 21K18255 (HK), 22H02845 (HK)], SENSHIN Medical Research Foundation (KM), Takeda Science Foundation (KM), KANAE Foundation for the Promotion of Medical Science (KM), The Uehara Memorial Foundation (KM), The Naito Foundation (KM), and Ohyama Health Foundation (KM).

## Conflict of Interest

The authors declare that the research was conducted in the absence of any commercial or financial relationships that could be construed as a potential conflict of interest.

## Publisher’s Note

All claims expressed in this article are solely those of the authors and do not necessarily represent those of their affiliated organizations, or those of the publisher, the editors and the reviewers. Any product that may be evaluated in this article, or claim that may be made by its manufacturer, is not guaranteed or endorsed by the publisher.
